# Moderate Chronic Treadmill Exercise Slows Dopaminergic Neuron Loss in a Rat Model of Parkinson's Disease and Alters RNA Content of Circulating Plasma Exosomes

**DOI:** 10.1002/jnr.70084

**Published:** 2025-10-13

**Authors:** Bruce A. Citron, Maynard Guzman, Bobak Shapdoor, Pranela Rameshwar, Arpine Sokratian, Amaan L. Shaikh, Anya E. Mausoof, Andrew B. West, Vedad Delic

**Affiliations:** ^1^ Laboratory of Molecular Biology, VA new Jersey Health Care System East Orange New Jersey USA; ^2^ Department of Pharmacology Physiology, & Neuroscience, Rutgers New Jersey Medical School Newark New Jersey USA; ^3^ Department of Medicine Rutgers New Jersey Medical School Newark New Jersey USA; ^4^ Neurobiology Department, Department of Pharmacology and Cancer Biology, Duke Center for Neurodegeneration Research Durham North Carolina USA; ^5^ Duke University School of Medicine Durham North Carolina USA

**Keywords:** alpha synuclein, CD63, chronic, exercise, exosomes, fibrils, parkinson's, preclinical, rat, treadmill

## Abstract

Exercise has been reported to improve outcomes in patients with Parkinson's disease, but the exact biological mechanisms remain incompletely understood. This study was conducted to determine whether chronic moderate treadmill exercise prevents dopaminergic neuron loss and Lewy body‐like inclusion burden in a preclinical model of PD. This study also sought to identify plasma exosome bound “exerkines” with potential to induce neuroprotective brain adaptations. Benefits of exercise are thought to be achieved in part through nucleic acids, lipids, and peptides termed exerkines abundant during exercise. These exerkines can induce cell‐specific exercise adaptations culminating in neuroprotection. Membrane‐free exerkines are subject to degradation in blood and may not effectively cross the blood‐brain barrier to induce brain adaptations to exercise, limiting their inter‐organ signaling potential. This led us to hypothesize that exerkines, bioactive RNAs in particular, may be selectively packaged into exosomes and released into plasma during exercise. Exosomes are small extracellular vesicles, selectively packaged and released by cells, with likely important differences in composition at rest, during injury and disease, and in response to exercise. Exosomes have been demonstrated to play a major role in inter‐organ communication. Chronic exercise was found to provide neuroprotection to dopaminergic neurons in the substantia nigra pars compacta, without affecting Lewy body‐like inclusion burden in the nigra or the striatum. Plasma exosomal RNA was isolated from age‐matched exercising and sedentary non‐PD rats and sequenced. We report 27 unique RNAs upregulated in the exosomes of exercisers, 3 mRNAs with clear neuroprotective potential.

**Summary**

- Exercise is known to slow PD progression, but the exact mechanisms by which this is accomplished are incompletely understood.
- We show that moderate exercise lasting for 1 month significantly slows the progression of PD in a preclinical rat model.
- We identify RNAs that are increased in plasma exosomes of rats undergoing moderate exercise. Exosomes are small, selectively packaged extracellular microvesicles that are known mediators of interorgan communication.
- They carry bioactive molecules, like the RNAs we identified, that have the potential to induce neuroprotective benefits of exercise.
- These exosomes may also facilitate the benefits of exercise in other neurological disorders.

## 1 Introduction

PD is a common neurodegenerative movement disorder affecting millions with well‐recognized motor deficits, and emerging recognition of associated non‐motor symptoms (Ahmad et al. 2023; Borgonovo et al. 2017). Genetic and environmental risk factors for PD have also been identified. Common genetic risk factors for PD include gain‐of‐function mutations in the leucine‐rich repeat kinase (LRRK2) (Taymans et al. 2023). gene and duplications or mutations in the gene coding for alpha‐synuclein (SNCA) (Magistrelli et al. 2021). In total, 23 risk genes (PARK 1–23) have been identified so far, with some having multiple genetic variants (Li et al. 2021; Tran et al. 2020). However, even the most common PD‐causing mutations account for only 1%–9% of all PD cases depending on the population (Pitz et al. 2024). In contrast, environmental risk factors for PD are far more common, including traumatic brain injury and exposure to herbicides, pesticides, and volatile toxicants (Delic et al. 2020). Regardless of the cause, PD treatments remain limited to symptom management using dopaminergic agonists and deep brain stimulation. While effective at managing symptoms, these treatments do not stop or slow the progression of PD.

Benefits of long‐term or chronic moderate exercise have been robustly associated with improved clinical outcomes for patients with PD (Tsukita et al. 2022). In a 6‐hydroxydopamine (6‐OHDA)–induced rat model of hemi‐parkinsonism, degeneration was alleviated with moderate exercise (Chen et al. 2018). Similar effects were reported in an adeno‐associated virus‐α‐synuclein (AAV‐α‐syn) rat model of PD (Crowley et al. 2018). However, bona fide Lewy body‐like inclusion formation, typical of PD, and progressive rather than acute neurodegeneration together with progressive transsynaptic spread of pathology are seen in the fibril model of PD (Volpicelli‐Daley et al. 2016). These aspects of human PD pathology are important when evaluating the benefits of exercise in animal models. Progressive neurodegeneration with transsynaptic spread of alpha synuclein pathology can be achieved with intracranial injection of preformed alpha synuclein fibril (PFF) (Volpicelli‐Daley et al. 2016). Using the PFF model of PD, we wanted to evaluate the effects of exercise on the progressive loss of dopaminergic neurons and Lewy body‐like inclusion burden. Moreover, we wanted to identify exercise factors abundant during exercise with the potential to induce neuroprotective adaptations.

Several lines of evidence indicate that circulating factors present in the blood may promote neurological restoration. Parabiosis between young and aged animals has been shown to confer improvements in behavioral function to aged animals (Castellano et al. 2016). In these studies, the circulatory systems of a young and aged animal are connected for a time and have shown improvements in various diseased states (Castellano et al. 2016). The feasibility of blood factors as potential treatments for neurological disorders was also demonstrated in humans, with the transfusion of plasma from young human donors to elderly recipients with mild cognitive impairment (Sha et al. 2019). Factors involved in the improvements could include exerkines that are released during or shortly after exercise by all tissues, with potential for autocrine and paracrine signaling. Common autocrine exerkines released during exercise with neuroprotective potential include BDNF and Musclin (Chow et al. 2022). Exerkines with endocrine effects on the brain are more controversial. For example, Irisin, a muscle‐derived peptide, is present in blood plasma with neuroprotective properties, but its direct link to brain tissue remains tenuous due to unresolved transcription issues and detection method reliability (Albrecht et al. 2020). Viral overexpression of irisin was shown to be neuroprotective in a mouse model of PD, but the study did not resolve the physiological irisin levels or mechanisms of action (Kam et al. 2022). Although the neuroprotective benefits of exercise are well‐known and largely irrefutable, the molecular mechanisms of exercise‐associated benefits remain incompletely understood (Chow et al. 2022). Better understanding of inter‐organ communication during exercise, particularly those with neuroprotective potential, can inform the development of future therapeutics.

Plasma exosomes are small (< 100 nm) extracellular micro‐vesicles, and their cargo includes selectively packaged, bioactive RNAs, proteins, and lipids protected from enzymatic breakdown in blood. Exosomes are facilitators of inter‐organ communication, are abundant after exercise, and can be readily isolated from blood. A recent human pilot study showed that exosomes present in the blood of healthy sedentary human subjects differ from those that undergo exercise in their micro‐RNA content (Garai et al. 2021) suggesting that those exercise‐specific exosomes may be involved in inter‐organ communication. A deeper understanding of neuroprotective factors in the blood that can reprogram brain cells toward regeneration would allow for the development of more specific and more effective treatments. Exosomes are a product of the endolysosomal pathway, synthesized and loaded with cargo, and then stored in multivesicular bodies (MVB) prior to release from the cell (Simons and Raposo 2009). They can be isolated from various biofluids, including blood, by several methods, such as differential centrifugation and filtration. Often, more than one method of isolation is employed to maximize the yield and purity. Exosomes contain membrane markers that prevent their phagocytosis in the blood, allowing distribution over long distances and reaching other organs (Parada et al. 2021). Exosomes do not elicit a strong immune response and have been shown to readily cross the blood‐brain barrier (Banks et al. 2020). Accepted exosomal biomarkers include CD9, CD63, and CD81 (Hoshino et al. 2020). Building on these studies, we wanted to determine the degree of treadmill exercise necessary to achieve neuroprotection in a preclinical PFF model of PD and to identify the peripheral exosomal RNA content released during neuroprotective levels of exercise. Changes in plasma exosomal RNA resulting from chronic exercise compared to sedentary control are currently absent from human and rat literature. This study will identify plasma exosome RNAs abundant at levels of moderate chronic exercise that can provide neuroprotection in PD rats. Results of this study will expand our understanding of exercise‐adaptive changes with neuroprotective potential.

We hypothesize that 1 month‐long moderate chronic exercise will slow the progression of PD as measured by dopaminergic neuron loss and Lewy body burden. Additionally, we hypothesize that plasma exosomal RNA cargo will have increased levels of mRNAs with the potential to induce neuroprotective brain adaptation to exercise.

## 2 Methods

### 2.1 Animal Usage

Male Sprague Dawley (SD) rats, 9–10 weeks of age, were obtained from Charles River and handled in accordance with the VA New Jersey Health Care System Institutional Animal Care and Use Committee (IACUC) guidelines. Rats were housed individually in standard polycarbonate 18‐quart tubs with bed‐o‐cob bedding, which was changed once a week. Rooms were kept at 22°C ± 4°C, and rats were kept 12 h on and 12 h off reverse light cycles to allow for experiments during rat subjects' active phases. To minimize stress during handling and experiments, rats were acclimated to the experimenter by handling for a week before experiments were performed. Two separate cohorts were used in this study. Cohort 1: nucleation of PD by injection of PFFs followed by exercise treatment and histology; cohort 2: exercise treatment followed by molecular biology to determine exosome content after exercise. All rats were kept on a standard *ad libitum* diet for food and water.

### 2.2 Recombinant αSyn PFF Synthesis and Injection

To initiate PD‐like pathology, rats were anesthetized and intracranially injected unilaterally into the anatomical right SNpc with 20 μg of PFFs or monomeric αSyn, as previously detailed by Delic et al. (Delic et al. 2022). The monomers and PFFs were generously provided by Dr. Andrew West at Duke University School of Medicine and were generated as previously described by Abdelmotilib et al. (Abdelmotilib et al. 2017). Briefly, recombinant mouse αSyn was expressed in *E. coli* , isolated, and purified using size exclusion chromatography and ion exchange, followed by endotoxin removal. αSyn was aggregated into fibrils using the aggregation assay (Polinski et al. 2018; Volpicelli‐Daley et al. 2014). Formed fibrils were fragmented by sonication and diluted to 5 μg/mL. A burr hole was made at X = 2.5, Y = 5.35–5.5, Z = 7.4–7.5 mm of Bregma and injected in a 4 μL volume over 30 min.

### 2.3 Exercise Protocol

Rats were placed on the treadmill facing away from the experimenter. Animals were continuously monitored while on the treadmill. Lanes were separated by plexiglass enclosers, preventing interactions between rats. The exercise group underwent a warm‐up for 5 min at 10 m/min and exercise for 30 min with no incline, at 20 m/min, 5 days a week. A 5‐lane MazeEngineers treadmill equipped with both a light signal and tail shock was used for exercise. Sedentary controls were present in the room during exercise. While the literature remains inconsistent on the speed that provides the most benefits, 20 m/min is tolerated for up to 45 min, 5 days a week (Lalanza et al. 2015). Up to 50% attrition was found at speeds greater than 20 m/min for 30 min. 20 m/min was used since PD rats consistently tolerated this speed with minimal attrition (1 rat).

### 2.4 Perfusion and Tissue Collection

Rats slated for IHC were deeply anesthetized and perfused with PBS, and then by 4% paraformaldehyde (PFA), followed by an overnight fixation in 4% PFA with agitation at 4°C. Brains were cryopreserved in 30% sucrose and stored at −80°C as previously described by Delic at al (Delic et al. 2022). Blood was drawn via left ventricle cardiac puncture and collected into a heparin‐lithium coated vacuum tube within 30 min of the last exercise. To obtain plasma, the blood was centrifuged twice at 8500 x g for 180 s using HemoCue StatSpin Express 2 Centrifuge to remove cells and cellular fragments. Plasma was kept on ice until the same day of exosomal isolation.

### 2.5 Statistics

Required sample size *n* = 10 was determined experimentally based on power analysis (expected sigma = 0.377, effect size = 1.33, significance 0.05, and power = 80%) for stereological estimation of TH+ neurons in the substantia nigra pars compacta (SNpc). *n* = 10 was used for comparing TH+ fiber density in the striatum. All histological groups had 2 controls (monomer, which was surgery vehicle control, and PFF injected positive control) and 1 experimental group, PFF + exercise. For molecular assays with higher sensitivity like RNA sequencing, *n* = 4 was determined experimentally to be adequate. Two groups: 1 sedentary control and 1 exercise experimental group. Rats were randomly assigned to each experimental or control group using a random number generator. Ordinary one‐way ANOVA test was used to determine significant differences in the TH+ neuronal counts in the SNpc. Significance was indicated by * *p* < 0.05 and ** *p* < 0.01. Closed circles represent individual data points, red error bars represent standard deviation, and black horizontal bar indicates mean. One‐way ANOVA with Tukey's multiple comparison post hoc results is now included in the figure caption for data where statistical analysis was performed; the entire ANOVA table and normality tests are included in supplemental Table S1 Data was analyzed and graphed using Prism for macOS. Rats were excluded from the study if they failed to exercise; 1 rat was replaced on the second day of exercise. No rats perished during the course of the study.

### 2.6 Immunohistochemistry

40‐μm thick serial sections were collected using a freezing microtome 1–4 through nigra and 1–6 through the striatum. The sections were stored in 50% glycerol with 0.1% sodium azide at −20°C. All tissue used for IHC underwent antigen retrieval for 30 min with agitation at 37°C in buffer (10 mM Citrate, 0.05% Tween‐20, pH 6.0). Blocking was done using serum of the species in which secondary antibodies were raised. For a list of all antibodies, please see Table 1. Tissue used for stereology was quenched in methanol with 0.6% hydrogen peroxide and probed with primaries raised against tyrosine hydroxylase (TH+) for dopaminergic neurons. Phosphorylated alpha synuclein (pSyn) at the serine 129 residue was also stained in the same way to visualize Lewy body‐like inclusions for manual counting. TH+ and pSyn were visualized with HRP‐conjugated secondary antibodies and DAB kit stain from Vector Labs. Tissue was counterstained with hematoxylin for nuclei. Stained sections were mounted on Fisherbrand Superfrost slides, dried, covered with Permount mountant, and covered with coverslips. For fluorescent staining, blocking was performed after antigen retrieval without methanol quenching, followed by incubation with fluorescent secondary antibodies Table 1. Fluorescent sections were counterstained with Hoechst and mounted on Superfrost slides, covered with ProLong Gold antifade mountant and covered.

**TABLE 1:** Primary and secondary antibodies used in this study.

Primary Antibody	Species	Type	Dilution	Vendor	Catalog #
TH	chicken	polyclonal	1 to 1000	Millipore	AB9702
NeuN	mouse	monoclonal	1 to 1000	Novus	NBP1‐92693
pSyn	rabbit	monoclonal	1 to 10,000	Abcam	ab51253
CD9	Rabbit	monoclonal	1 to 1000	Fisher	PIMA531980
CD63	Rabbit	polyclonal	1 to 1000	Fisher	PIPA592370
CD81	Rabbit	monoclonal	1 to 1000	Fisher	PIMA532333
FNDC5	Rabbit	polyclonal	1 to 1000	Invitrogen	PIPA579279
Anti BDNF	Rabbit	monoclonal	1 to 1000	Abcam	NC1089906
NPY	Rabbit	Monoclonal	1 to 1000	Cell Sginal	77100SF
Protein Controls					
Irisin			1 ng	PhoenixPharm	NC1884506
BDNF			1 ng	R&D Systems	248‐BD‐005
Secondary Antibody					
Alexa 488 anti‐rabbit	goat	polyclonal	1 to 1000	Invitrogen	A32721
Alexa 555 anti‐chicken	goat	polyclonal	1 to 1000	Invitrogen	A21437
Alexa 647 anti‐mouse	goat	polyclonal	1 to 1000	Invitrogen	A32728
IRDye 680rd anti‐rabbit	donkey	polyclonal	1 to 10,000	LICOR	925–68,073
IRDye 800CW anti‐mouse	donkey	polyclonal	1 to 10,000	LICOR	926–32,212
HRP anti‐rabbit	goat	polyclonal	1 to 1000	Cell Signaling	7074P2
HRP anti‐mouse	goat	polyclonal	1 to 2000	Invitrogen	62–6520

### 2.7 Unbiased Stereological Quantification of DNs

Sections used for counting cover the entire SNpc with 1 in 4 sampling and equal 120 μm spacing, with random frame placement through all areas counted. Guard zone height was set to 2 μm. Sampling grid size (e.g., 200 μm) was adjusted as needed, due to lesion size variability, averaging 5 objects counted at each of 100 to 200 x‐y locations. TH+ positive DAB‐stained dopaminergic neurons and TH‐ neurons were counted. Dopaminergic neurons were differentiated from non‐dopaminergic neurons based on dark brown staining of soma. TH‐ neurons were differentiated from glia by their large size Figure 2C and from TH+ neurons by lack of DAB staining.

### 2.8 Confocal Microscopy

Sections were imaged in four channels with an Olympus FluoView 3000 confocal microscope operating Fluoview FV31S‐SW software. Excitation wavelengths of 405 nm, 488 nm, 561 nm, and 640 nm were optimized in each channel before image acquisition. Z‐stack images for visualization of pSyn, TH, and NeuN were obtained at 20× and 60× magnification using the same optimized laser settings. All images were exported and processed to MaxZ projections with ImageJ 1.53a (Schneider et al. 2012).

### 2.9 Li‐Cor Scanning

A LI‐COR Odyssey CLx scanner was used to measure TH+ fiber density in the striatum to evaluate loss of TH+ fibers, which is concomitant with neuron loss in the ipsilateral PFF‐injected nigra. Sections on Fisherbrand Superfrost Plus Microscope slides were scanned tissue side down without a coverslip, with an offset of 0.00 mm and a 21 μm resolution. The area of capture was set using LI‐COR software. Signal intensity was normalized to the contralateral side.

### 2.10 Exosomal Isolation

Exosomes were isolated from fresh rat plasma with either ultracentrifugation, the exo‐quick exosome isolation kit (Fisher Health catalog# NC1404477), or the Total Exosome Isolation (from plasma) kit (Invitrogen, catalog #4484450) according to manufacturers' instructions. Exosomal precipitation kits from Fisher or Invitrogen resulted in a far higher yield compared to ultracentrifugation Figure 2S. Briefly, 1.5 mL of fresh rat plasma from each sample was aliquoted equally into two 1.5 mL tubes (750 μL per tube). The plasma samples were centrifuged at 2000 x g for 20 min at room temperature to eliminate cells and debris. The supernatant was then transferred to a new tube and centrifuged at 10,000 x g for 20 min to completely remove debris. The clarified plasma, approximately 700 μL per tube, was then transferred to a new tube and placed on ice. To precipitate exosomes, 0.5 volumes of PBS (350 μL) were added and vortexed to mix thoroughly, followed by 0.2 volumes (210 μL) of exosome precipitation reagent. The solution was mixed by vortexing and incubated at room temperature for 10 min. The sample was then centrifuged at 10,000 x g for 10 min at room temperature. The supernatant was discarded, and the precipitated exosomes were centrifuged again at 10,000 x g for 1 min. The supernatant was carefully discarded, and each exosome pellet was resuspended in 100 μL of PBS. Two tubes containing exosome suspensions from the same sample were combined in one tube to obtain a final volume of 200 μL exosome suspension per sample. The exosome suspension was stored at 4°C overnight and was processed for RNA extraction within 24 h.

### 2.11 Exosome Validation

Plasma exosomes isolated by either exosome isolation kits or differential centrifugation were assessed for the total number of particles of 100 nm size on a Nanosight. Particle yield with a diameter < 100 nm was increased by 10,000‐fold using exosome isolation kits. For further validation, samples underwent immunoblotting for exosomal markers CD 9, CD 61, CD 83 Figure S1 with CD 63 showing a robust signal in exercise and sedentary combined samples Figure 3B.

**FIGURE 1:**
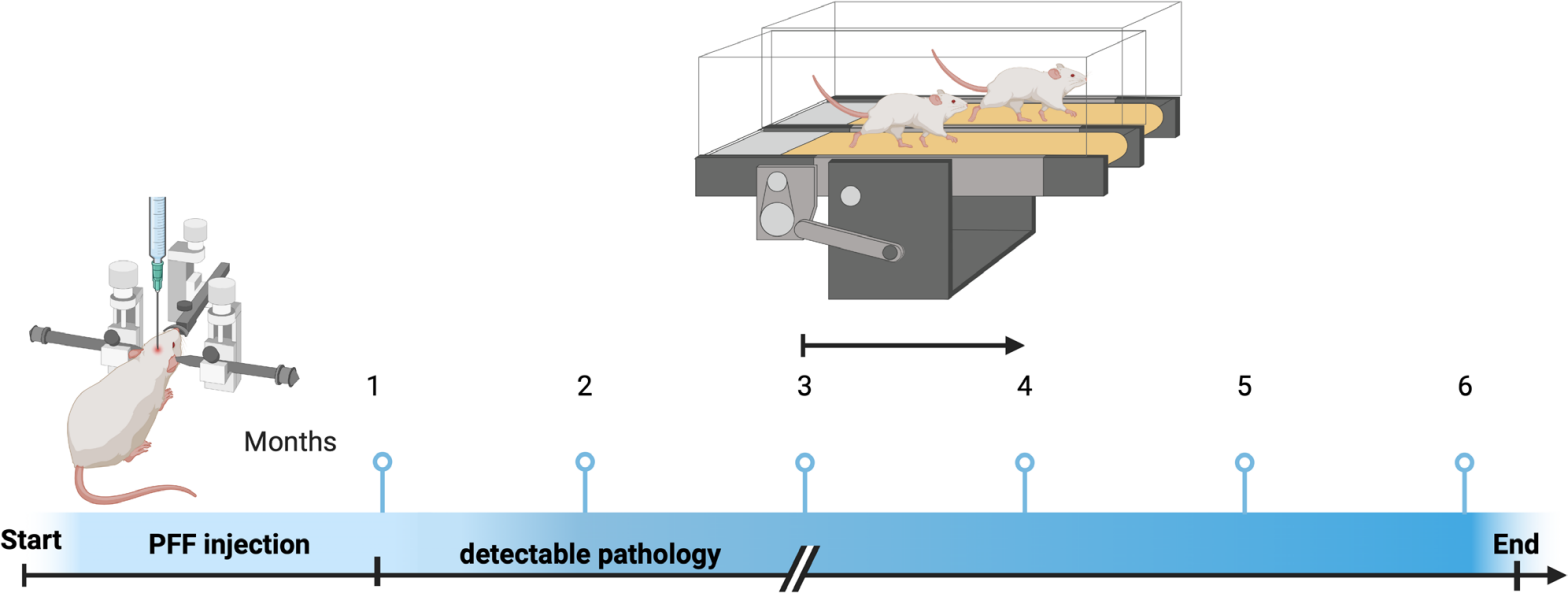
Experimental design and timeline. Month‐long moderate exercise was initiated 3 months after PD nucleation, and the experimental endpoint was 6 months after PFF injection. Created in BioRender. Delic, V. (2025) https://BioRender.com/wsdp6nd.

### 2.12 Westerns to Validate Exosomes and Detect Neuroprotective Peptides

Total protein was isolated using RIPA buffer from exercising and sedentary rat plasma exosomes. The exosome homogenate was vortexed, and cell debris was spun down at 4°C for 5 min at 14,000 rpm. Protein concentration was determined with the Pierce BCA Protein Assay Kit and iMark Absorbance Microplate Reader (Bio‐Rad). For western blot analysis, up to 30 μg of protein was loaded onto the 4%–20% Mini‐PROTEAN TGX Stain‐Free Gels. Electrophoresis was conducted for 30 min at 200 V. For total protein normalization, before transfer, the stain‐free gels were cross‐linked with a Bio‐Rad ChemiDoc imaging system, with a UV transilluminator for 1 min. The gel was transferred on a Bio‐Rad Trans‐Blot Turbo, and Trans‐Blot Turbo RTA Mini 0.2 μm PVDF for 7 min at 2.5 A and 25 V. The membrane was subsequently scanned for total protein on the ChemiDoc, washed 2× with TBST, and blocked for 1 h in 5% fat‐free powdered milk reconstituted in TBST. Blots were washed three times and incubated overnight in primary antibodies followed by HRP‐linked secondaries for 2 h Table 1. The blots were visualized with Clarity Max Western ECL substrate and imaged on the ChemiDoc. Analysis was performed using Image Lab Version 5.2.

### 2.13 RNA Isolation

RNA was extracted from exosomes using the Total Exosome RNA and Protein Isolation kit (Invitrogen, catalog #4478545) according to the manufacturer's instructions. An equal volume of pre‐warmed 2X Denaturing solution was added to 200 μL of exosome suspension, mixed by vortexing, and incubated on ice for 5 min. 400 μL of Acid‐Phenol:Chloroform was added to this mixture, the tubes were vortexed to mix thoroughly, and then centrifuged at > 10,000 x g at room temperature for 5 min. The upper aqueous phase was transferred to a new tube, and the transferred volume noted (~300 μL). 1.25 volumes of 100% ethanol (375 μL) were added to the aqueous phase and mixed by vortexing. The mixture was then applied to the Filter Cartridge, centrifuged briefly at 10,000 x g, the flow‐through discarded, and washed with Wash solutions three times as indicated in the protocol. Finally, RNA was eluted in a fresh tube by applying 27 μL of nuclease‐free water pre‐warmed to 42°C to the filter cartridge, incubating at room temperature for 1 min, and then collecting the eluate by centrifugation. A second eluate was similarly collected from the same filter cartridge to ensure full recovery of RNA. Eluted RNA was analyzed on a TapeStation using high sensitivity RNA ScreenTape.

### 2.14 RNA Sequencing

Due to the picogram amounts of RNA, RNA‐Seq libraries were prepared using Takara SMARTer Stranded Total RNA‐Seq Kit v3—Pico Input Mammalian (Takara Bio USA Inc. catalog #634485). 1 ng of extracted RNA was used as input for library construction without fragmentation. Illumina adapters and indexes were added after first strand synthesis and amplified using 10 PCR cycles. The product was purified with NucleoMag beads at a 1.0X bead ratio. This was followed by depletion of the ribosomal cDNA, RNA‐seq library amplification, and purification. The purified libraries were analyzed with D1000 ScreenTape on TapeStation and stored at −20°C. The libraries were quantified by Qubit fluorometer, pooled in an equimolar ratio, and sequenced on a NovaSeq X Plus instrument.

## 3 Results

### 3.1 Moderate Exercise Slows PFF Induced Neurodegeneration

**FIGURE 2:**
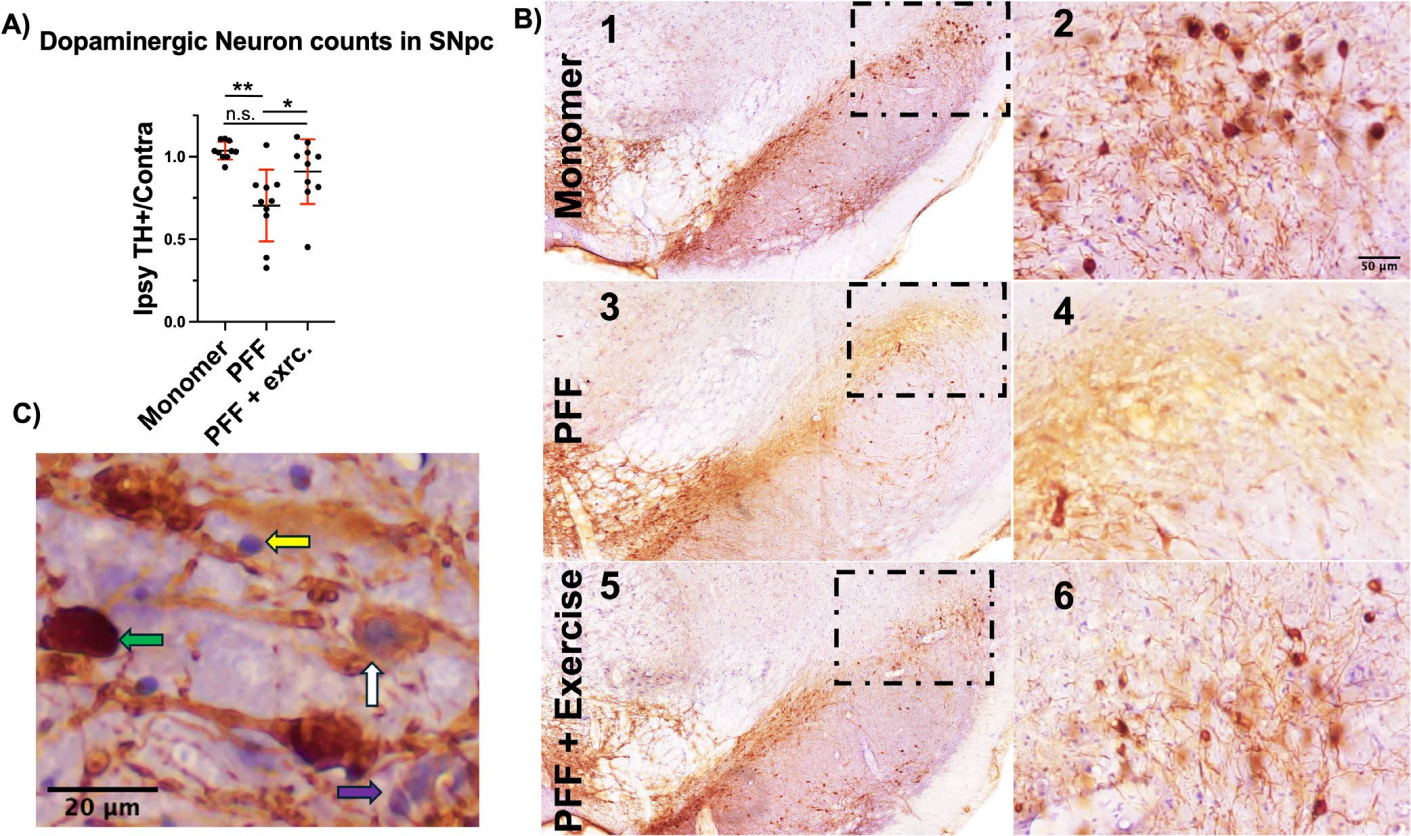
Stereological estimation of dopaminergic neurons in the SNpc normalized to the contralateral side. Chronic exercise slowed PFF induced dopaminergic neuron loss compared to sedentary PFF controls A. Representative images of ipsilateral injected nigra with an inserted digital zoom, vehicle monomer injected B1, B2, sedentary PFF injected B3, B4, and PFF with exercise B5, B6. Representative image indicating dopaminergic neurons staining strongly for TH+ green arrow, and weakly staining for TH+ white arrow and non‐TH+ neurons purple arrow, distinguished by large nucleus and nonneuronal cells yellow arrow distinguished by small nucleus C. Representative image demonstrating ability to discriminate between dopaminergic neurons strongly green arrow, and weakly staining for TH+ white arrow and non‐TH+ neurons purple arrow, and nonneuronal cells yellow arrow in (Figure 2C). ANOVA: F (2, 27) = 9.60, *p* < 0.001. Tukey's multiple comparisons test adjusted *p* value: Monomer vs. PFF *p* < 0.001, Monomer vs. PFF + exercise *p* = 0.238 n.s., PFF vs. PFF + exercise *p* < 0.032. Mean and standard deviation: Monomer = 1.037, 0.054, PFF = 0.704, 0.217, PFF+ exercise = 0.910, 0.196.

One month of daily moderate exercise, beginning 3 months after PD was initiated Figure 1, was found to significantly reduce neurodegeneration in rats undergoing moderate exercise compared to sedentary controls Figure 2. DN loss in the sedentary group was 33% and 13% in the exercise group Figure 2A. Representative images of DNs in the SNpc of monomer, PFF, and PFF + exercise (Figure 1B).

### 3.2 Denervation of the Striatum Is Not Affected by Moderate Month‐Long Exercise at the 6‐Month Endpoint

**FIGURE 3:**
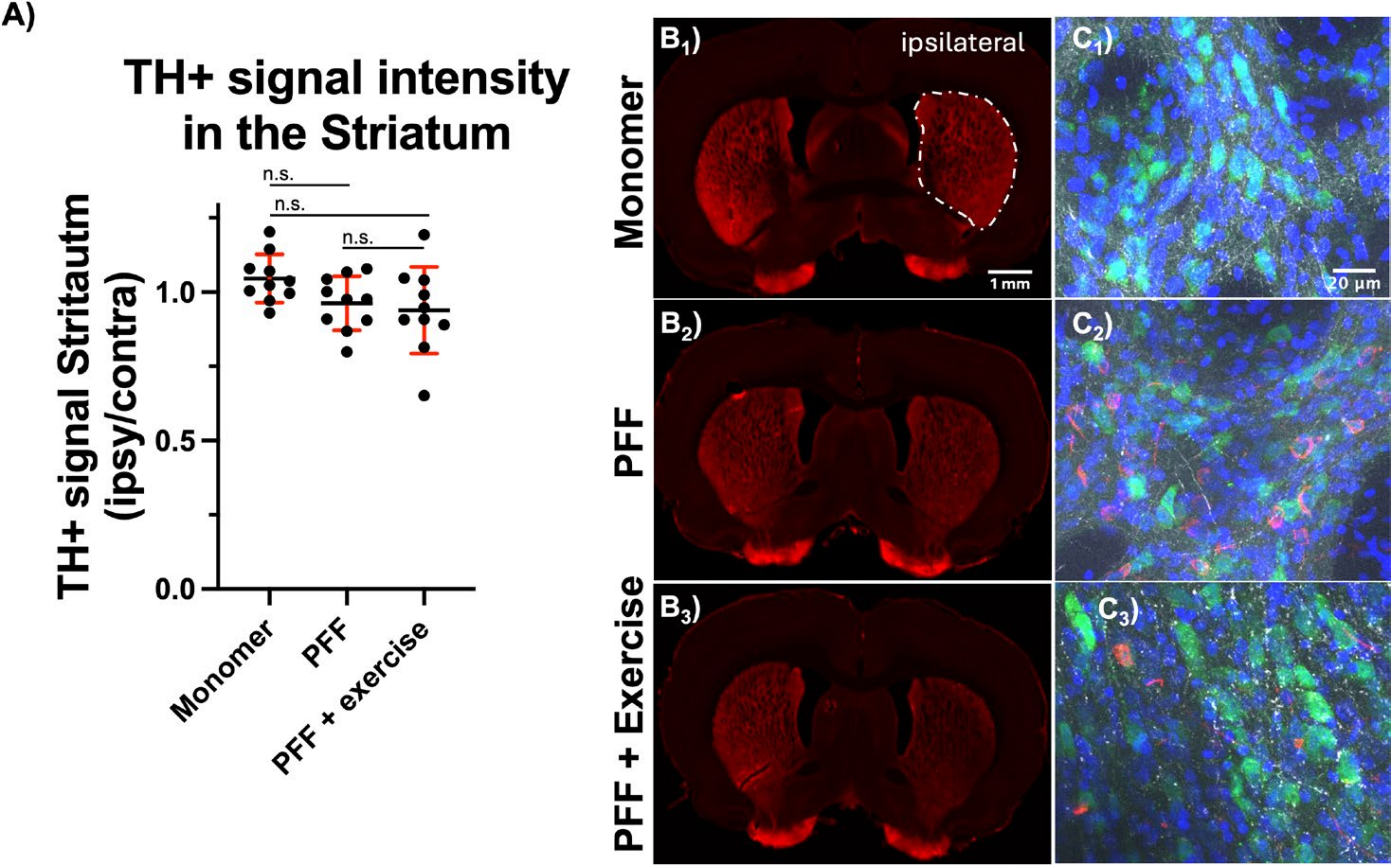
Quantification of TH+ fiber density signal in the ipsilateral striatum. Striatum ipsilateral to the injection showed a small reduction in TH+ fiber density, that was not improved by exercise A. Representative images of the TH+ fiber in the Str of Monomer B1, PFF B2, and PFF + exercise B3. High magnification representative images of Str ipsilateral to the SNpc injection, Monomer C1, PFF C2, and PFF + Exercise C3. TH+ fibers (white), nuclei (blue), NeuN (green), pSyn (red), side ipsilateral to SNpc injection (white arrow). White dashed line indicates area quantified. ANOVA: F (2, 27) = 2.62, *p* = 0.091. Tukey's multiple comparisons test adjusted *p* value: Monomer vs. PFF *p* = 0.223, Monomer vs. PFF + exercise *p* = 0.094, PFF vs. PFF + exercise *p* = 0.884. Mean and standard deviation: Monomer = 1.046, 0.082, PFF = 0.962, 0.091, PFF + exercise = 0.939, 0.146.

Denervation of the striatum was quantified by measuring TH+ signal intensity of fiber projections in the striatum. Mean denervation (decrease in signal ipsilateral to the injection, normalized to the contralateral un‐injected side) was 8.0% between monomer and PFF and 10.23% between monomer and PFF + exercise group Figure 3A. Differences were not significant. Representative images of TH+ signal in the striatum with the *right* side of coronal sections ipsilateral to the injection in SNpc (Figure 3 B1‐B3). Representative images of pathology in the striatum ipsilateral side, LB and Lewy neurites *red*, NeuN+ cells green, Hoechst nuclear stain blue, and TH+ fibers white (Figure 3 C1‐C3).

### 3.3 Moderate Exercise Does Not Affect Lewy Body‐Like Inclusion Burden in Nigra or Striatum

**FIGURE 4:**
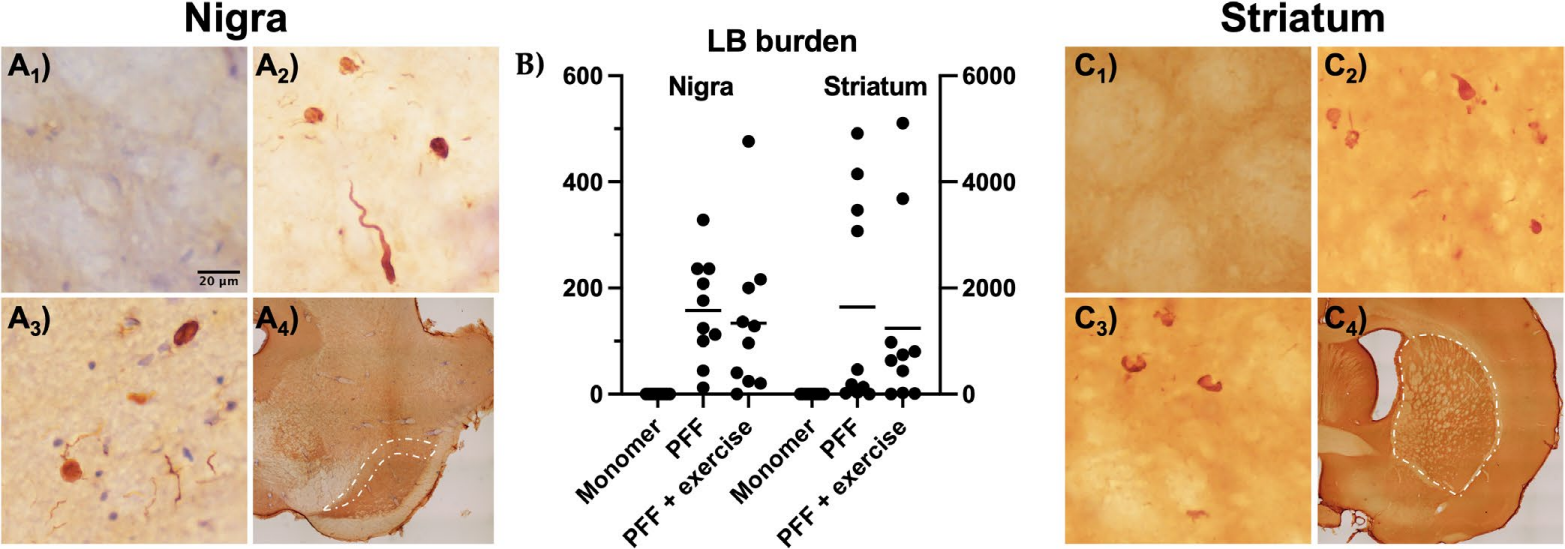
Lewy Body‐like inclusion burden quantification in the Nigra and the Str. Representative images of SNpc probed with antibody against pSyn in monomer A1, PFF A2, and PFF + Exercise A3. Representative image indicating anatomical location of the substantia nigra pars compacta quantified A4. Quantification of LB in the SNpc and the Str in monomer, PFF, and PFF + exercise rats B. Representative higher magnification images are shown of the Str probed with antibody against pSyn in monomer C1, PFF C2, and PFF + Exercise C3. Representative image indicating anatomical location of the striatum quantified C4. Assumptions of the ANOVA test not met, data failed normality test, and statistical analysis not performed. Mean LB burden in nigra: Monomer = 0, PFF = 158, PFF + exercise = 134. Mean LB burden in striatum: Monomer = 0, PFF = 1642, PFF + exercise = 1242.

Representative images of Lewy body‐like inclusions (LB) and neurites absent in vehicle control (Figure 4 A1) and present in PFF injected ipsilateral SNpc of sedentary (Figure 4 A2) and exercised rats (Figure 4 A3). LB burden in nigra can reach up to 476 per rat brain *left* (Figure 4B) and up to 5106 in striatal medium spiny neurons right (Figure 4B). Representative images of LB and neurites absent in vehicle control (Figure 4 C1) and present in PFF injected ipsilateral Str of sedentary (Figure 4 C2) and exercised rats (Figure 4 C3).

### 3.4 Exosomes Released During Moderate Exercise Contain mRNA With Neuroprotective Potential

**FIGURE 5:**
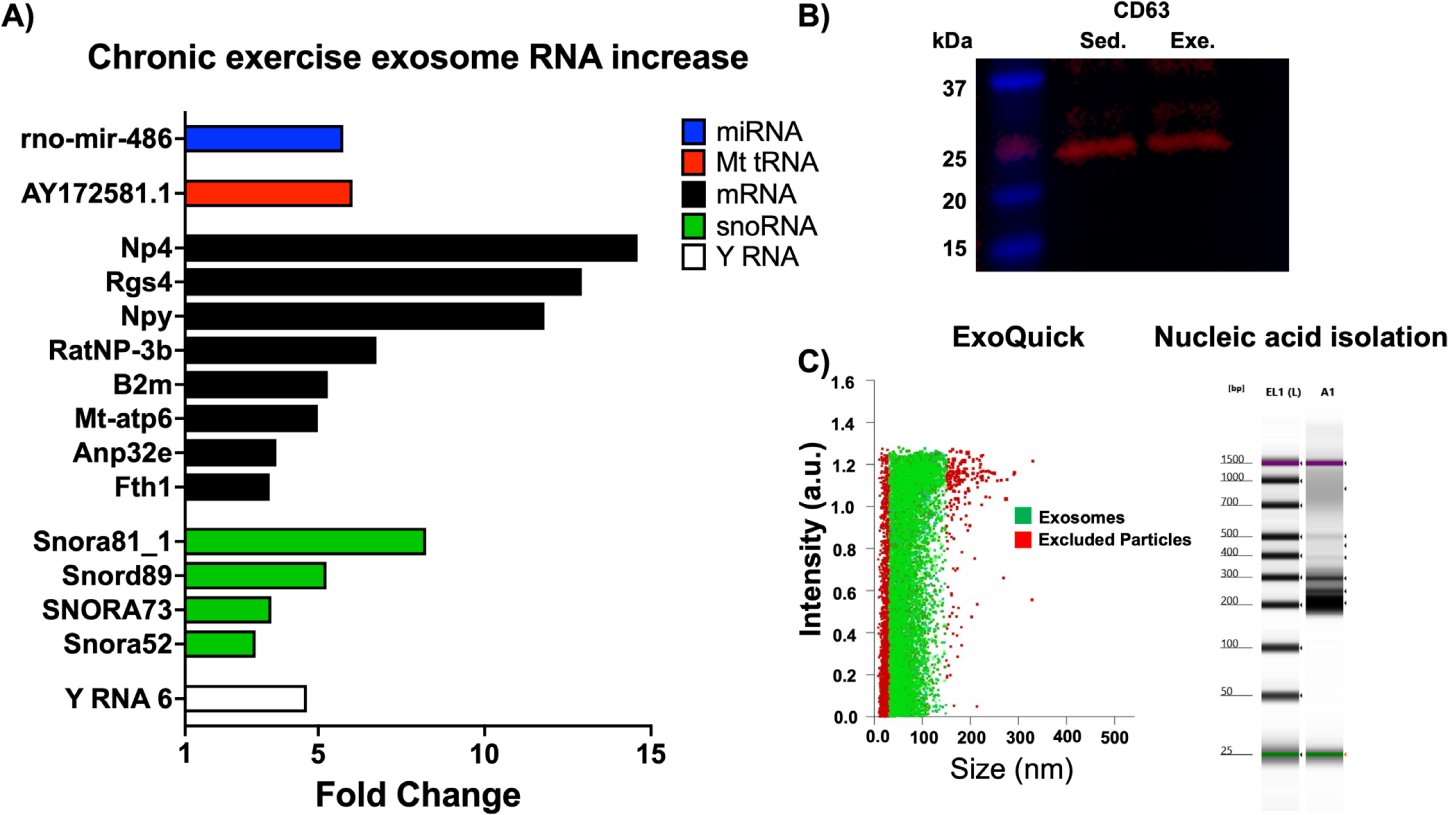
Upregulation of exosomal RNA in plasma exosomes of exercisers compared to sedentary controls. Fold increase in plasma exosome RNAs with known function A. Immunoblot of sedentary and exercised combined rat samples for exosomal marker CD 63 B. Quality control for isolated exosome yield and nucleic acid isolated from those exosomes C. Bars indicate fold change differences of exercisers compared to sedentary controls.

27 unique RNA molecules were significantly upregulated with fold change ranging from 3 to 142 Table 2. Representation of RNA by type was: 4% micro‐RNA, 7% mitochondrial RNA, 37% mRNA, 33% small nucleolar RNA, 15% small nuclear RNA, and 4% Y RNA Table 2. Fold changes for Rgs4, Npy, and Mt‐atp6 mRNAs were 13, 12, and 5, respectively Figure 5A. Exosomes isolated with ExoQuick were used for sequencing over ultracentrifugation due to a much higher yield Figure 5C, Figure 2S. Exosomes were also validated with Western blot for CD 63 present in both sedentary and exercise samples Figure 5B. Irisin, NPY, and BDNF proteins were not detected in sedentary or exercise‐derived exosomes Figure 3S.

**TABLE 2:** All RNAs upregulated in exerciser plasma exosomes.

Name	Chrom.	Max group mean	FC	*P*‐value	ENSEMBL	Biotype
rno‐mir‐486	16	9.66	5.75	0.02	ENSRNOG00000062490	miRNA
AY172581.23	MT	10.82	80.97	9.23E‐03	ENSRNOG00000033957	Mt_tRNA
AY172581.1	MT	10.72	6.03	4.00E‐02	ENSRNOG00000029070	Mt_tRNA
Np4	16	12.96	14.6	1.13E‐03	ENSRNOG00000069755	protein_coding
Rgs4	13	7.72	12.92	1.18E‐03	ENSRNOG00000002773	protein_coding
Npy	4	5.67	11.8	3.76E‐04	ENSRNOG00000046449	protein_coding
RatNP‐3b	16	32.56	6.75	0.01	ENSRNOG00000038135	protein_coding
gene:ENSRNOG00000063835	5	6.03	6.73	7.94E‐03	ENSRNOG00000063835	Protein_coding
B2m	3	8.59	5.29	5.70E‐03	ENSRNOG00000017123	protein_coding
Mt‐atp6	MT	12.05	4.99	0.02	ENSRNOG00000031979	protein_coding
Anp32e	2	7.28	3.74	0.03	ENSRNOG00000021168	protein_coding
Fth1	1	7.27	3.54	0.04	ENSRNOG00000022619	protein_coding
Pf4	14	5.05	3.09	0.05	ENSRNOG00000028015	protein_coding
LOC120095472	10	6.19	25.48	2.35E‐03	ENSRNOG00000053534	snoRNA
LOC120095739	11	7.32	13.27	1.40E‐03	ENSRNOG00000060035	snoRNA
Snora81_1	10	53.47	8.24	1.77E‐03	ENSRNOG00000060944	snoRNA
LOC120102095	3	6.14	6.76	0.02	ENSRNOG00000052201	snoRNA
LOC120098679	19	25.64	6.74	3.84E‐03	ENSRNOG00000053462	snoRNA
Snord89	9	6.34	5.25	3.00E‐02	ENSRNOG00000055019	snoRNA
LOC120095490	10	20.31	4.19	5.00E‐02	ENSRNOG00000063269	snoRNA
SNORA73	14	76.18	3.59	0.03	ENSRNOG00000052548	snoRNA
Snora52	1	43.27	3.12	4.00E‐02	ENSRNOG00000065361	snoRNA
LOC120093427	6	9.31	142.31	5.04E‐03	ENSRNOG00000059869	snRNA
LOC120096085	12	6.78	105.97	5.50E‐03	ENSRNOG00000071022	snRNA
LOC120101155	2	5.5	6.29	3.00E‐02	ENSRNOG00000062782	snRNA
LOC120098346	18	453.31	3.25	0.04	ENSRNOG00000066317	snRNA
Y_RNA_6	4	143.7	4.66	0.01	ENSRNOG00000060430	Y_RNA

## 4 Discussion

Exercise has been shown to improve outcomes in patients with PD and in animal PD models. Identifying inter‐organ communication with the potential to effect these neuroprotective changes is therefore a critical step toward informing the development of neuroprotective therapeutics. Since exosomes are selectively packaged and released during exercise, this study sought to identify exosomal RNA content at levels of exercise that achieve neuroprotection. Month‐long moderate treadmill exercise was reported to achieve neuroprotection in a 6‐OHDA toxicant‐induced model PD (Garcia et al. 2017). The highest tolerable exercise intensity was determined by titration. While most rats could run faster than 20 m/min, for every additional increase of 5 m/min, there was attrition of 2 or more animals. We found equal attrition among rats regardless of whether they underwent intracranial PFF injection. 20 m/min was achievable by nearly all rats for 5 days a week for 30 min. There was an initial weight loss of up to 10% in each animal that normalized by the second week of exercise, and the weights between former exercisers and sedentary rats were indistinguishable by the end of the study.

### 4.1 Moderate Exercise Slows Neuronal Loss in SNpc but Does Not Affect the LB Burden

Dopaminergic neuron loss was abated by chronic moderate exercise Figure 2, while LB burden was not affected Figure 4. LB burden was expected to decrease with improved neuronal survival since LB pathology can be used to stage the progression of PD. There are a couple of possible explanations for this observation. By 3 months, pathogenic molecular fragments of PFFs “seeded” all the nearby neurons, setting them on a path toward LB formation and eventual degeneration. In this case, LB formation and maturation may be inevitable, but the impact of its formation may be mitigated by trophic signaling in response to exercise. While the total burden may not be impacted, LB ultrastructural composition may be different in response to exercise, making their formation less toxic. Heterogeneity of LB ultrastructural composition has been reported between synucleinopathies that also differ in their rate of progression and disease severity (Tarutani et al. 2022). For example, multiple system atrophy advances more rapidly than PD and has a distinct banding pattern from dementia with LB and Parkinson's disease dementia. It is possible that exercise may alter LB formation processes within PD, thereby limiting their cytotoxicity. Treadmill exercise (5 m/min for 30 min, 6 days for 2 months) was shown to decrease inclusion burden and dopaminergic neuron loss in young A53T transgenic mice injected bilaterally into the striatum with PFFs (Dutta et al. 2024). This neuroprotection was shown to be PPARα dependent, and the presence of the A53T alpha synuclein mutation, striatal PFF injection, and age of mice may have influenced the more robust responsiveness to exercise.

In our previous studies using this model, we found that while loss of dopaminergic neurons correlates with denervation of the striatum, the ratio is not proportional (Abdelmotilib et al. 2017; Delic et al. 2022). One possible explanation for this is that surviving neurons compensate by reinnervating the striatum to provide dopamine. PD symptoms in patients do not appear until dopaminergic neuron loss in nigra exceeds 85%, suggesting that similar compensation may be underway in humans.

### 4.2 Fold Increase in Multiple RNA Types in Plasma Exosomes of Chronic Exercisers

Total abundance and metabolic profile of plasma exosomes among active men has been shown to be similar at rest and after exercise (Darragh et al. 2023). In a pilot study of marathon runners, circulating micro‐RNAs were found upregulated in plasma (Kuji et al. 2021). We therefore wanted to identify RNAs in plasma exosomes of age‐matched rats undergoing a level of exercise that provided neuroprotection in PD. Isolated exosomes were validated by their size using a NanoSight particle analyzer Figure 5C and by surface markers CD9, CD63, and CD81 (Lin et al. 2015). Only CD63 was detected at 25 and 65 kDa bands Figure 5B, Figure S1. Treatment with irisin, an exerkine peptide, was recently reported to attenuate inflammasome activation in PD, ameliorating the PFF‐induced pathology (Zhu et al. 2025). We therefore wanted to determine if irisin and other previously reported neuroprotective peptides, neuropeptide Y and BDNF, were present in exosomes of exercisers. The peptides were not detected in exosomal protein extract using immunoblotting Figure 3S. Absence of irisin, NPY, and BDNF peptides or their mRNAs from plasma exosomes of exercisers in our study suggests that they either reach the brain unaided by microvesicles or may be synthesized in the brain during exercise.

Neuropeptide Y, Mt‐atp6, Rgs4, and Rno‐mir‐486 were increased by 12, 5, 13, and 5‐fold, respectively, in plasma exosomes of chronic exercisers Figure 5A. NPY protein levels decrease in the brains of patients with PD (Duarte‐Neves et al. 2016), increase in the brain with exercise (Chen et al. 2007) and have been shown to provide some level of neuroprotection (Pain et al. 2022). Mt‐atp6 RNA codes for mitochondrially encoded ATP synthase membrane subunit, ATP synthase F_0_ subunit a or 6, that serves as a proton channel essential in driving oxidative phosphorylation (Das et al. 2023; Nijtmans et al. 1995). Mutations in the gene coding for Mt‐atp6 contribute to multiple neurological mitochondrial disorders (Kenvin et al. 2022). Mt‐atp6 abundance in exosomes of otherwise healthy exercisers is speculative but may be upregulated in response to increased global energy demands by tissues during exercise and may indirectly benefit distal neuronal tissue not directly involved with peripheral exertion during exercise. Rgs4 is a controversial modulator of dopaminergic neuron survival, where its inhibition or knockout protects dopaminergic neurons (Lerner and Kreitzer 2012), but low levels of Rgs4 mRNA and protein are found in postmortem brains of schizophrenia patients (Bakker et al. 2007; Mikdache et al. 2021). Rgs4 regulates signaling and heart rate in the sinoatrial node, and its expression is elevated in cardiac tissue during excessive exercise (Cifelli et al. 2008; Rogers et al. 1999). Rno‐mir‐486, elevated in exerciser plasma exosomes, is a micro‐RNA enriched in muscle tissue (Samani et al. 2022), suggesting that a significant proportion of the exosomes collected within 30 min after exercise in chronic exercisers may be muscle‐derived.

### 4.3 Neuroprotective Muscle‐Brain Cross Talk

Accumulating evidence indicates that skeletal muscle releases signaling molecules “myokines”, with the potential to affect the brain (Chow et al. 2022). These messengers could affect the brain indirectly through adipose tissue by adiponectin, or the liver via insulin‐like growth factor 1. Also, and of most interest herein, are molecules released by muscle cells with the potential to directly affect the brain. We think that the direct effect of muscle on the brain can be achieved through exosomes released during prolonged levels of moderate physical activity. From our exosome RNA data Figure 5A, we see a 5.75‐fold increase in rno‐mir‐486 RNA, implicating skeletal muscle origin. mir‐486 RNA is essential for normal skeletal muscle function and is abundant in healthy muscle tissue (Samani et al. 2022). A 12‐fold increase in exosome NPY mRNA may increase BDNF. NPY treatment of cortical neurons in vitro was shown to increase BDNF mRNA and protein levels (Croce et al. 2013). BDNF has well‐established neuroprotective and anti‐inflammatory properties (Xu et al. 2017). The abundance of Mt‐atp6 mRNA discussed above suggests that exosomes may increase oxidative phosphorylation and thereby ATP production in the brain. Together, these findings suggest that exosomes abundant after moderate exercise in plasma of chronic exercisers are enriched for exosomes from muscle tissue, and that these exosomes may provide neuroprotection by modulating anti‐inflammatory, pro‐survival, and energy production signaling.

### 4.4 Study Limitations

This study had 5 male rats in each of the exosomal RNA sequencing groups. While informative and significant, the study would be improved by the addition of female rats as PD outcomes differ in men and women. In this study, rats underwent moderate chronic exercise for 1 month, and longer studies may uncover additional benefits, or at least a point of diminishing returns where, with additional exercise, no further benefits are achieved. Additionally, the effect of exercise on alpha‐synuclein inclusion formation and composition was not evaluated and should be investigated in future exercise PD studies.

### 4.5 Future Direction and Translational Potential

Follow‐up studies should determine whether exercise prior to PD nucleation can provide improved neuroprotection. To ascertain whether plasma exosomes can induce neuroprotective exercise adaptations in the brain directly, intracerebroventricular (ICV) delivery of exercise exosomes to PD rats should be performed. This would unambiguously answer the questions of their neuroprotective properties directly on the brain. Next, intravenous infusion of exercise donor exosomes should be tested to determine the degree to which IV delivery can achieve neuroprotection compared to ICV. In vitro studies using human iPSC‐derived dopaminergic neurons and PFFs can determine cell‐specific effects of exercise exosomes on dopaminergic neurons in PD. In vitro studies can also determine the individual contribution of identified exosomal RNAs. These studies would provide preliminary data for eventual human testing of exercise‐derived exosomes or delivery of identified exosomal RNA with the potential to induce neuroprotective exercise adaptations. With 33 clinical trials of exosome therapies underway for various diseases (Rezaie et al. 2022), there exists a clear path for eventual clinical application of exercise‐derived exosomes and their content.

## Author Contributions

V.D. developed the study and directed the project, B.A.C. developed the study, provided RNA seq. expertise, M.G., B.S., A.E.M., and V.D. performed experiments; A.S. generated figures and performed analysis; P.R. provided exosome expertise and use of equipment for exosome validation; A.S. and A.B.W. generously produced, validated, and provided PD‐inducing PFFs.

## Conflicts of Interest

The authors declare no conflicts of interest.

## Supporting information


**Data S1:** Supporting Information

**Figure S1:** Immunoblot of CD9, CD69, and CD81, exosomal markers.

**Figure S2:** Particle characterization of ultracentrifugation exosomal yield compared to ExoQuick. Particle yield after ultracentrifugation **A**. Particle yield after ExoQuick isolation **B**. Nucleic acid yield from exosomes isolated with ExoQuick **C**.

**Figure S3:** Immunoblots of NPY, irisin, and BDNF, peptides associated with exercise and neuroprotection.

**Table S1:** jnr70084‐sup‐0004‐Table.xlsx.**: Table 1 S: Supplemental statistical analysis: normality test, and ANOVA results**. Statistical results are shown for the various parameters observed including the tyrosine hydroxylase (TH+) features and LB. Substantia nigra (nigra) and striatum (str) are included.

## Data Availability

The data that support the findings of this study are openly available upon reasonable request and online in the NCBI Gene Expression Omnibus (GEO) repository, accession GSE302532.
