# Supplementary figures and images for: Moderate Chronic Treadmill Exercise Slows Dopaminergic Neuron Loss in a Rat Model of Parkinson's Disease and Alters RNA Content of Circulating Plasma Exosomes
Source: J Neurosci Res. 2025 Oct 13;103(10):e70084. doi: 10.1002/jnr.70084 (PMC12516300; doi:10.1002/jnr.70084)

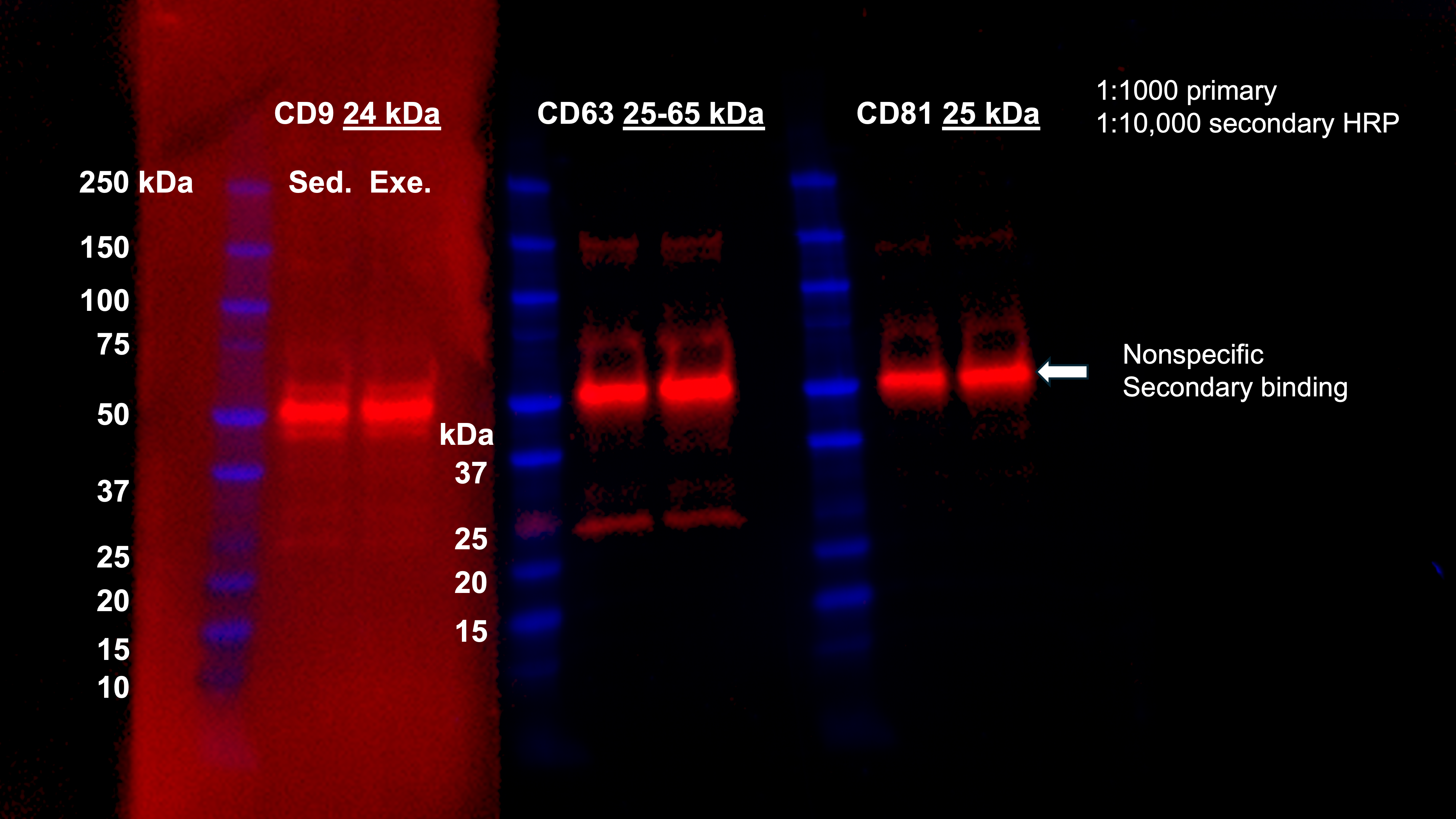

Supplement: Supplementary file 2 — Figure S1: Immunoblot of CD9, CD69, and CD81, exosomal markers. [file JNR-103-e70084-s003.tiff]

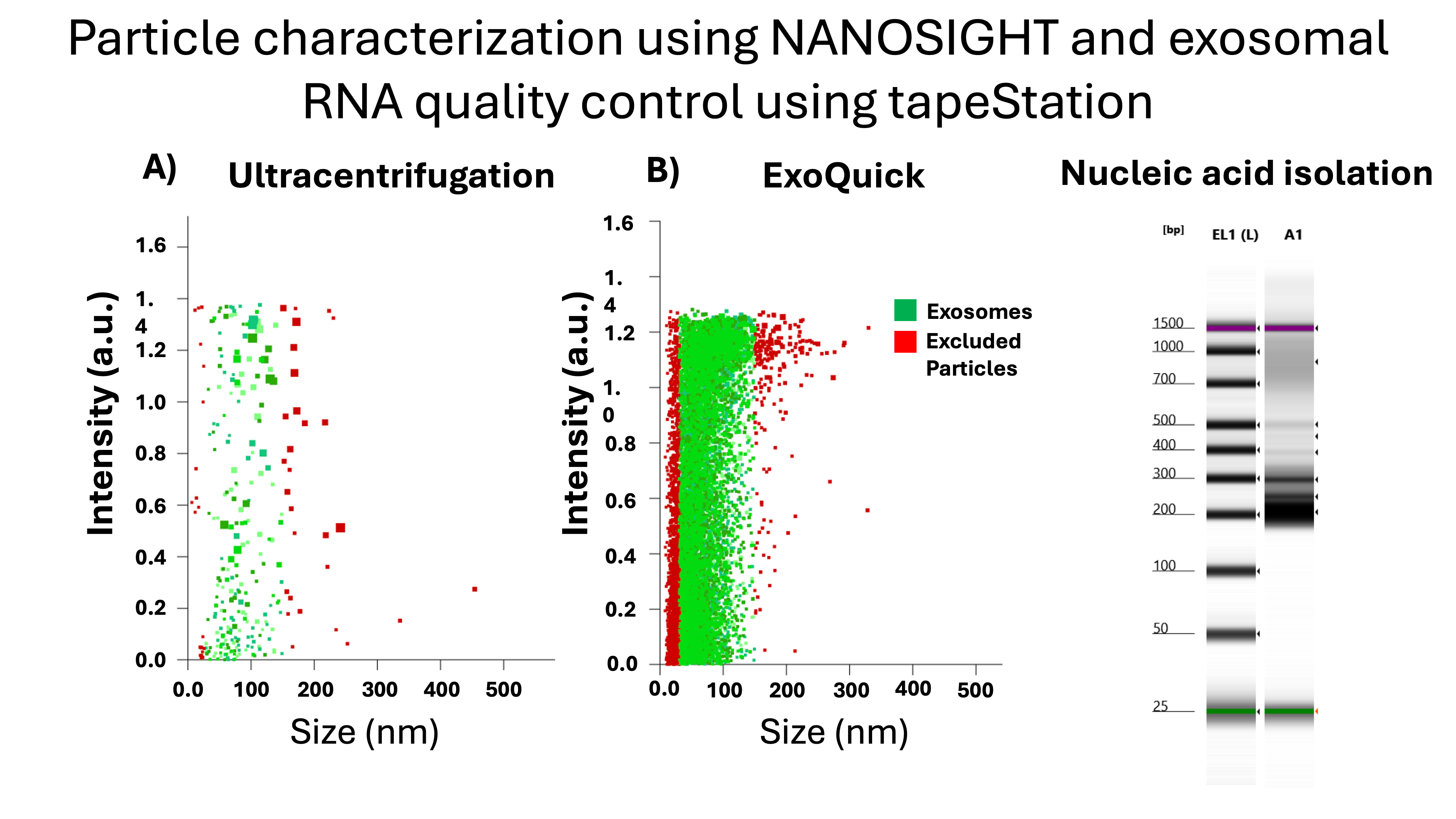

Supplement: Supplementary file 3 — Figure S2: Particle characterization of ultracentrifugation exosomal yield compared to ExoQuick. Particle yield after ultracentrifugation A. Particle yield after ExoQuick isolation B. Nucleic acid yield from exosomes isolated with ExoQuick C. [file JNR-103-e70084-s002.tiff]

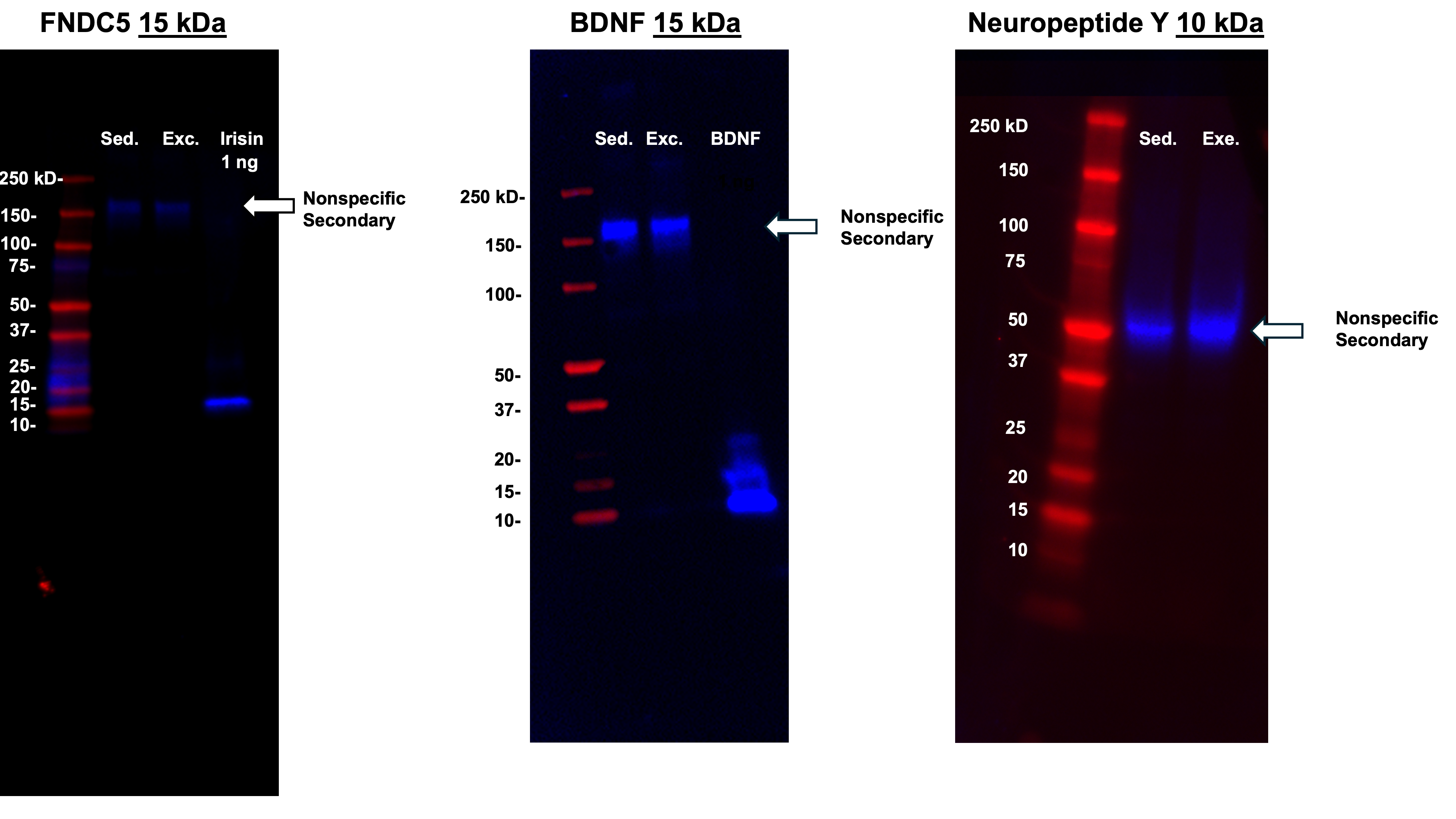

Supplement: Supplementary file 4 — Figure S3: Immunoblots of NPY, irisin, and BDNF, peptides associated with exercise and neuroprotection. [file JNR-103-e70084-s001.tiff]
